# Comparative Metagenomics of Anode-Associated Microbiomes Developed in Rice Paddy-Field Microbial Fuel Cells

**DOI:** 10.1371/journal.pone.0077443

**Published:** 2013-11-01

**Authors:** Atsushi Kouzuma, Takuya Kasai, Gen Nakagawa, Ayaka Yamamuro, Takashi Abe, Kazuya Watanabe

**Affiliations:** 1 School of Life Sciences, Tokyo University of Pharmacy and Life Sciences, Hachioji, Tokyo, Japan; 2 Graduate School of Science & Technology, Niigata University, Niigata, Japan; Wilfrid Laurier University, Canada

## Abstract

In sediment-type microbial fuel cells (sMFCs) operating in rice paddy fields, rice-root exudates are converted to electricity by anode-associated rhizosphere microbes. Previous studies have shown that members of the family *Geobacteraceae* are enriched on the anodes of rhizosphere sMFCs. To deepen our understanding of rhizosphere microbes involved in electricity generation in sMFCs, here, we conducted comparative analyses of anode-associated microbiomes in three MFC systems: a rice paddy-field sMFC, and acetate- and glucose-fed MFCs in which pieces of graphite felt that had functioned as anodes in rice paddy-field sMFC were used as rhizosphere microbe-bearing anodes. After electric outputs became stable, microbiomes associated with the anodes of these MFC systems were analyzed by pyrotag sequencing of 16S rRNA gene amplicons and Illumina shotgun metagenomics. Pyrotag sequencing showed that *Geobacteraceae* bacteria were associated with the anodes of all three systems, but the dominant *Geobacter* species in each MFC were different. Specifically, species closely related to *G. metallireducens* comprised 90% of the anode *Geobacteraceae* in the acetate-fed MFC, but were only relatively minor components of the rhizosphere sMFC and glucose-fed MFC, whereas species closely related to *G. psychrophilus* were abundantly detected. This trend was confirmed by the phylogenetic assignments of predicted genes in shotgun metagenome sequences of the anode microbiomes. Our findings suggest that *G. psychrophilus* and its related species preferentially grow on the anodes of rhizosphere sMFCs and generate electricity through syntrophic interactions with organisms that excrete electron donors.

## Introduction

Plant photosynthesis fixes atmospheric carbon dioxide to produce organic matter that serves as the primary carbon and energy sources for heterotrophic organisms, including humans. Recently, a novel concept has emerged for the on-site conversion of photosynthesized organic matter into electricity [1-3]. To cite an instance, Strik et al. [1] developed a pot-culture microbial fuel cell system (plant MFC) to generate electric power from organics excreted from the roots of reed mannagrass. A similar system was applied for energy production from cultured rice plants [2]. Furthermore, on-site electricity generation in a rice paddy field was demonstrated using a sediment-type MFC (sMFC), which consisted of graphite anodes and cathodes set in the rice rhizosphere and flooded water, respectively [3]. Using this system, 15 mW m^-2^ of electricity (normalized to the anode projection area) was generated in a sunlight-dependent manner [4]. Evidence suggests that the rice paddy-field rhizosphere sMFC functions as an ecological solar cell, in which plant photosynthesis is coupled to the microbial conversion of organics into electricity and has the potential to provide an energy source for on-site monitoring of environmental parameters during rice-plant cultivation [5].

 To better understand how electricity is generated from plant-root exudates, rhizosphere microbes associated with the anodes of sMFCs have been characterized [6,7]. Molecular analyses of microbes associated with the anodes of rice pot-culture MFCs have detected *Desulfobulbus*-like species, members of the family *Geobacteraceae*, and as yet uncultured representatives of the domain *Archaea* [6]. Examination of anode-associated rhizosphere bacterial communities in reed mannagrass sMFCs has revealed that several species of *Geobacteraceae*, which include electrochemically active bacteria (EAB), were located on root surfaces, but were more abundant on the graphite granular anodes [7]. Notably, anaerobic cellulolytic bacteria affiliated with the families *Clostridiaceae* and *Ruminococcaceae* were abundant in areas where most EAB were found, suggesting that the current was generated via the hydrolysis of cellulose. Putative short-chain fatty acid-utilising facultative denitrifiers affiliated with the families *Rhodocyclaceae* and *Comamonadaceae* were also detected, and were speculated to be the major competitors of EAB for electron-donating organic material.

 Preliminary sequencing analyses of rice paddy-field sMFCs have indicated that bacterial sequence types affiliated with the order *Rhizobiales* and family *Beijerinckiaceae* are present in anode-associated rhizospheres [3], while it has still been unclear what kinds of EAB are present in rice paddy-field sMFCs. To deepen our understanding of the rice paddy soil microbiomes that are associated with electricity generation in sMFCs, here, we conducted comparative metagenomic analyses of anode-associated microbes in three MFC systems: an sMFC installed in a rice rhizosphere, and acetate- and glucose-fed MFCs, in which pieces of graphite felt that had functioned as anodes in a rice paddy-field sMFC were used as microbe-bearing anodes. The microbiomes established in each system were compared by pyrotag sequencing of 16S rRNA gene amplicons and shotgun metagenomics. In particular, we focused our analyses on putative EAB affiliated with the family *Geobacteraceae*, as members of this family have been demonstrated to generate electricity in bioelectrochemical systems [8,9].

## Materials and Methods

### Rice paddy field-rhizosphere sMFCs

An area of the Egawa rice paddy field owned by Noda Natural Symbiotic Farm Co. (Noda, Chiba, Japan) was used for sMFC experiments. For the farm-land use, specific permissions were not required, since the study was conducted according to the request from the owner company. The field studies did not involve endangered or protected species. The configuration of the sMFC system was fundamentally similar to that described previously [5]. In the present study, a single electrode system was set for one rice-plant hill. The anode and cathode were made of graphite felt (3 mm in thickness, GF-80-3F, Sohgoh Carbon) and had projection areas of approximately 550 cm^2^. The electrode had a single hole (10 cm in diameter) in the center to allow planting of the rice plants. A platinum catalyst (TEC10E20A, Tanaka Kikinzoku Hanbai; 0.1 mg cm^-2^) was loaded on the graphite felt cathode using nafion as a binder. The anode and cathode were connected via epoxy-encapsulated wires, and the circuit was completed using an external resister (1000 Ω). The voltage (*E* [V]) across the resister was automatically monitored every 10 min using a data logger (HA-1510, Graphtec). Rice-plant seedlings (*Oryza sativa* L. cv. Koshihikari) were transplanted on April 30, 2011 and cultivated using standard procedures [3], after which the voltage was monitored.

### Single-chamber MFCs

To construct the single-chamber MFCs, the graphite-felt anodes from rice-paddy sMFCs producing a stable current were recovered along with the surrounding soil to be used as anodes and provide a source of microbial inocula in the MFC reactor. Before being inserted into the reactor, a 3 × 10 cm piece of the rice paddy-field MFC anode was shaken in sterile water to remove loosely attached soil particles. The MFC reactors were single-chamber cylindrical reactors that contained air cathodes (approximately 20 cm^-2^, 0.5 mg platinum cm^-2^, four polytetrafluoroethylene layers), as described previously [10,11]. The reactor was filled with 450 ml electrolyte solution (pH 7), which contained (per liter) KCl (0.1 g), NH4Cl (0.2 g), NaH2PO4•2H2O (3.1 g), and Bacto yeast extract (0.1 g). Prior to MFC operation, oxygen in the electrolyte solution was removed by bubbling with pure nitrogen gas. The anode and cathode were connected with electric wires and an external resistor (1 or 10 kΩ), and the voltage across the resistor was measured using a voltage data logger. After the initial drop in voltage, either sodium acetate or glucose (0.5 g liter^-1^) was added to the electrolyte solution as a growth substrate.

### Polarization analyses

Current (*I* [A]) was calculated using the equation; *I* = *E*/*R*, where *E* (V) is the cell voltage and *R* (Ω) is the resistance, and current density (*J*, mA cm^-2^) was calculated based on the projected anode area. Polarization curves were plotted using a potentiostat (HSV-100, Hokuto Denko) and were then used to estimate cell-performance indices (open-circuit voltage [*V*_oc_], short-circuit current density per projection area of the anode [*J*_sc_], and maximum power density per projection area of the anode [*P*_max_]) [12].

### Pyrotag sequencing of 16S rRNA gene amplicons

DNA was extracted from bulk soil (0.5 g) or anode graphite-felt pieces (0.5 × 0.5 cm) for use as template for pyrotag sequencing. Briefly, soil particles loosely attached to the rice paddy-field rhizosphere anode were first removed by shaking in sterile pure water. DNA was then extracted using a FastDNA SPIN Kit for Soil (Q-Bio) according to the manufacturer's instructions, except that bovine serum albumin was added at 0.1 mg ml^-1^, and purified DNA was finally dissolved in 50 μl of the DES solution supplied in the kit. PCR amplification of 16S rRNA gene fragments (V1-V3 region) was performed using primers ad-tag-8F (5’-CGTATCGCCTCCCTCGCGCCATCAGXXXXXXGAGTTTGATCMTGGCTCAG-3’) and ad-533R (5’-CTATGCGCCTTGCCAGCCCGCTCAGTTACCGCKRCTGCTGRCAC) [13], in which the underlined sequences were adaptors added for pyrosequencing and XXXXXX was an arbitrary tag sequence for sample identification [14]. The PCR mixture and thermal cycling conditions were as described previously [15]. Amplicons were purified using a QIAquick PCR purification kit (Qiagen) and mixed at a concentration of 1 ng μl^-1^ for each amplicon. The mixed amplicons were subjected to pyrosequencing using a Genome Sequencer FLX system at the Dragon Genomics Center (Mie, Japan), and phylogenetic analyses were conducted using the DDBJ 16S rRNA database (Feb. 21, 2012) and the BLASTN program [16] with an RDP classifier [17]. An operational taxonomic unit (OTU) was defined as a unique sequence or group of sequences with homologies of over 97%. Rarefaction analysis was performed using the ESPRIT program [18]. Sequence alignments were conducted using CLUSTAL W (ver. 1.83) [19] and program MEGA (ver. 5) [20], and phylogenetic trees were constructed by the neighbor-joining method [21]. To evaluate the robustness of the inferred tree, the bootstrap resampling method [22] was used with 100 replicates.

### Shotgun metagenome sequencing

DNA was extracted from 4 g of bulk rice paddy field soil and 3-cm^2^ pieces of the graphite-felt anodes from the rice paddy-field sMFC, and the acetate- and glucose-fed MFCs. Large soil particles attached onto the sMFC anode were removed by washing with filtered pure water before DNA was extracted. Total genomic DNA was extracted using a FastDNA SPIN kit for Soil, and the DNA quality was evaluated by agarose-gel electrophoresis, spectophotometeric analysis using the NanoDrop system (Thermo Scientific), and Quant-iT dsDNA BR Assay kit (Invitrogen). Approximately 5 µg of quality-checked DNA from each reactor anode was used for library construction and sequence analyses. High-throughput sequencing of the metagenome samples was performed in the Takara Dragon Genomics Center using one lane of the HiSeq 2000 sequencing system (Illumina). For each sample, an indexed paired-end DNA library was prepared using a NEBNext DNA Library Prep Reagent set for Illumina (New England Biolabs) and a TruSeq DNA Sample Preparation v2 kit (Illumina). The quality of generated libraries was evaluated using a 2100 Bioanalyzer (Agilent). Cluster generation was performed using a TruSeq PE Cluster v3 kit (Illumina). The libraries were sequenced from both ends for 100 cycles using a TruSeq SBS Kit v3 (Illumina). Approximately 4 gigabases (Gb) of metagenomic data were generated from each sample (details are described in Table 1) and used for subsequent analyses.

**Table 1:** Summary of numerical data for metagenomic analyses of the four samples.

Sample name	Source	No. of read	Total read length (bp)	No. of contig	Average contig length (bp)	Total lengh of contig (Mb)	No. of predicted gene^*a*^	Average gene length (bp)	No. of BLAST- hit gene^*b*^
Bulk soil	Bulk soil in the rice paddy field	35,762,974	3,576,297,400	99,946	199	19.89	54,073	177.3	7,887
Anode-associated soil	Soil associated with an anode of rice paddy-field sMFC	44,532,578	4,453,257,800	81,997	191	15.66	79,799	170.2	10,987
AM-anode biofilm	Anode biofilm in acetate-fed MFC	38,511,356	3,851,135,600	45,459	1,314	59.73	91,548	560.8	35,567
GM-anode biofilm	Anode biofilm in glucose-fed MFC	45,231,880	4,523,188,000	25,572	2,682	68.58	79,070	728.1	35,716

a A number of genes predicted in contigs by the MetaGeneAnnotator program.

b A number of predicted genes showing similarity to sequences in the NCBR-NR database (e-value ≤ 1.0 × 10^-5^ in the BLASTP analysis).

### Metagenome informatics

Sequence reads (100 bp each) were assembled into contigs using MetaVelvet software (version 1.2.01) [23]. MetaGeneAnnotator software [24] was used to predict potential protein-coding regions (predicted genes) in contigs. Predicted gene sequences were translated into protein sequences using NCBI Genetic Code 11 (http://www.ncbi.nlm.nih.gov/Taxonomy/Utils/wprintgc.cgi). BLASTP in BLAST+ software [25] was used to align translated protein sequences to the NCBI-NR database (downloaded on February 2013 from ftp://ftp.ncbi.nlm.nih.gov/blast/db/) using an e-value of 10^-5^ unless otherwise stated, and the resulting BLASTP files were analyzed according to NCBI taxonomy using MEGAN software (version 4.70.4) [26] with the following LCA parameters: Min score, 50; top percent, 10.0; Min support, 5. The KEGG viewer module in MEGAN was used to map the sequences in each sample to KEGG pathways [27]. Tetranucleotide BLSOM was also used for the taxonomic assignment of metagenome contigs [28,29].

Database deposition

Nucleotide sequences determined in the present study have been deposited into the NCBI Short Read Archive database under accession number: DRA001011.

## Results and Discussion

### Electric outputs from rhizosphere sMFCs in the rice paddy-field

**Figure 1:**
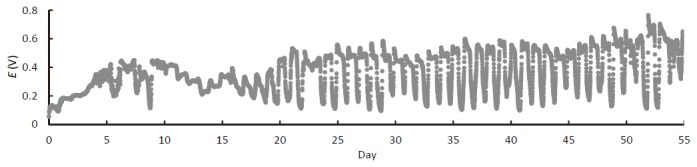
Representative time course of cell voltage for a rice paddy-field sMFC.

The electric output (*E*) from the rice paddy-field sMFC was characterized by circadian oscillation (Figure 1), indicating that the electricity generation was photosynthesis-dependent [3]. Approximately 20 days of operation were required for the sMFC to reach a stable circadian oscillation, likely owing to the growing time needed for the rice roots to contact the graphite felt anode. The observed circadian oscillation of the electric output indicates that the electricity was mainly generated from organic matter excreted from the roots of rice plants.

**Figure 2:**
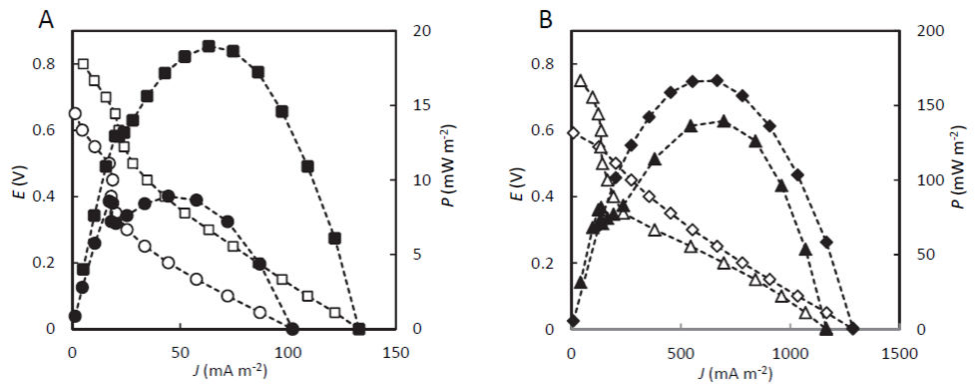
Polarization (open symbols) and power (closed symbols) curves showing the fuel-cell performances. (A) Rice paddy-field sMFCs in the daytime on days 15 (circles) and 50 (squares) of operation. (B) Single-chamber MFCs fed acetate (diamonds) or glucose (triangles).

Polarization analyses (Figure 2A) indicated that the rice paddy-field sMFC exhibited higher fuel-cell performance in the daytime (on day 50) than before the circadian oscillation was apparent (day 15). *V*_oc_, *J*_sc_, and *P*_max_ of the rice paddy-field sMFC on day 50 were 0.78 ± 0.05 V (mean ± SD, n = 5), 132 ± 25 mA m^-2^, and 19 ± 3.2 mW m^-2^, respectively, which are similar to the values reported previously for similar sMFCs [4]. The polarization curves for rhizosphere sMFCs were biphasic, characterized by a steep-decline phase in the high-voltage region (above 0.4 V), followed by a moderate-decline phase (Figure 2A). This trend was also observed in rhizosphere sMFCs set at different rice hills. Similar polarization curves were observed previously for reed mannagrass sMFCs [1]. Although the cause of the biphasic polarization curve is not clear, it is possible that the catabolism of substrates with different redox potentials by microbes with different catabolic activities would lead to the observed electrical output profiles.

### Electric output from MFCs using rhizosphere anodes

It has been reported that plants exhaust a number of photosynthesized organic compounds, predominantly organic acids and sugars, from roots [30,31]. Our previous study found that rice plants excrete glucose and acetate as major root exudates, and that the supplementation of rhizosphere sMFC anodes with acetate enhances electricity generation [3]. Based on these results, here, we constructed MFCs using anodes obtained from the rhizosphere sMFCs and added supplemental acetate or glucose to further enrich for the anode-associated, electricity-generating microbes. After initiating the operation, the electric outputs from the acetate-fed and glucose-fed MFCs rapidly increased and became stable (Figure S1). EAB were further enriched on the anodes of these MFCs by changing the electrolyte solutions when the electric outputs dropped.

Polarization analyses of the acetate-fed and glucose-fed MFCs were conducted several hours after the electrolyte solutions were replaced, and representative curves (day 242) are shown in Figure 2B. The *P*_max_ value (mean ±SD, n = 3) for the acetate-fed MFC was 161 ± 15 mW m^-2^ and that of the glucose-fed MFC was 146 ± 7 mW m^-2^, which were 7- to 9-fold higher (based on the projected anode area) than the power densities observed for the rhizosphere sMFCs (day 50). These results suggest that EAB were substantially enriched on anodes in both MFC reactors.

Interestingly, the glucose-fed MFC displayed a biphasic polarization curve that was similar to those of the rhizosphere sMFCs (Figure 2A), but such a curve was not observed for the acetate-fed MFCs. We speculated that the types of EAB and the electricity-generating mechanisms of the glucose-fed MFCs were similar to those in the rhizosphere sMFCs.

### Phylogenetic analyses of 16S rRNA pyrotag sequences

**Figure 3:**
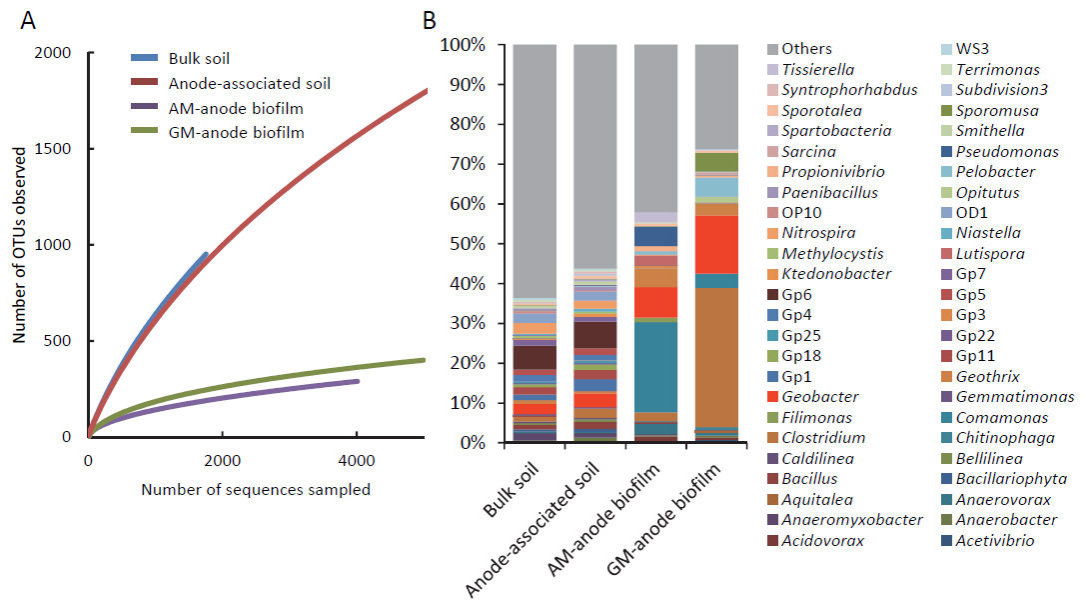
Analyses of pyrotag-sequencing data for bacterial communities. (A) Rarefaction curves showing the diversity of OTUs (similarity cut off of 95%). (B) Relative abundances of major genus-level bacterial groups estimated using the RDP classifier. Detailed data are shown in Table S2.

To compare the phylogenetic compositions of bacteria associated with anodes of the sMFC and acetate- and glucose-fed MFCs, pyrotag sequencing of 16S rRNA-gene amplicons [14] was conducted using DNA samples obtained from anode-associated soil of rhizosphere sMFCs (anode-associated soil), and the anode biofilms of acetate-fed MFCs (AM-anode biofilm) and glucose-fed MFC (GM-anode biofilm). Bulk soil of the rice paddy field (bulk soil) was also analyzed as a control. Comparisons of the overall OTUs detected in the samples indicated that the enrichment of anode-associated bacteria in these different MFC reactors resulted in a marked decrease (less than 25%) of phylogenetic diversity, as shown in the plotted rarefaction curves (Figure 3A). Concerning the total community structure at the genus level (Figure 3B), the bulk soil and anode-associated soil displayed similar profiles, in which *Acidobacteria* subgroup Gp6 was the most abundant, representing 6% of the total bacteria in these samples (see Table S1 for details). Consistent with a previous report for soil samples [32], a large number of uncultured *Acidobacteria* subgroups were also detected. *Geobacter* was also detected abundantly in these soil samples, representing approximately 2% and 3% of the total bacteria in the bulk and anode-associated soil, respectively. The ratios of *Geobacteraceae* sequences were previously reported to have increased from 0.13% to 0.74% in response to an increase in current density in a rhizosphere sMFC [7]. Here, *Geobacter* sequences were also more abundant in anode-associated soil than in bulk soil, suggesting *Geobacter* species were involved in electricity generation in the rhizosphere sMFCs.

The structures of bacterial communities were substantially altered after anode-associated microbes of sMFCs were grown in the acetate- and glucose-fed MFCs (Figure 3B). In AM-anode biofilms, *Comamonas* was abundant, composing 22% of the total bacteria. In a previous study [33], members of the genus *Comamonas* were detected in acetate-fed MFCs, and a *Comamonas* strain capable of generating electricity from acetate was identified. Molecular analyses of microbiomes in the rhizosphere sMFCs revealed that sequences of the family *Comamonadaceae* were abundant, suggesting that these oxygen- and/or nitrate-respiring organisms competed with EAB for electron donors [7]. In the acetate-fed MFC, both tasks of *Comamonas* need to be considered. However, because *Comamonas* was not detected in the anode-associated soil, it is not likely that these bacteria substantially affected electricity generation in the rhizosphere sMFC.

In GM-anode biofilms, members of the genus *Clostridium* were the most abundant, representing 33% of the total detected bacteria (Figure 3B). This genus includes a variety of carbohydrate-fermenting organisms [33], and members of *Clostridium* and related species are frequently detected in MFCs fed carbohydrates as fuels [34-36]. It has also been reported that syntrophic interactions between *Clostridium* and *Geobacter* enable electricity generation from cellulose [37]. These observations suggest that organisms represented by the identified *Clostridium* sequences fermentatively degraded glucose in the glucose-fed MFC reactor.

Possible EAB affiliated with the genus *Geobacter* were increased in the AM- and GM-anode biofilms (7% and 14% to the total bacteria, respectively; details are discussed below) (Figure 3B). In addition, members of the genus *Geothrix* [38] were also increased in these samples (Table S1). The increased ratios of these *Geobacter* species in the AM- and GM-anode biofilms likely corresponded to the increased power and current densities of the MFC reactors compared to those of the rhizosphere sMFCs (Figure 2).

**Figure 4:**
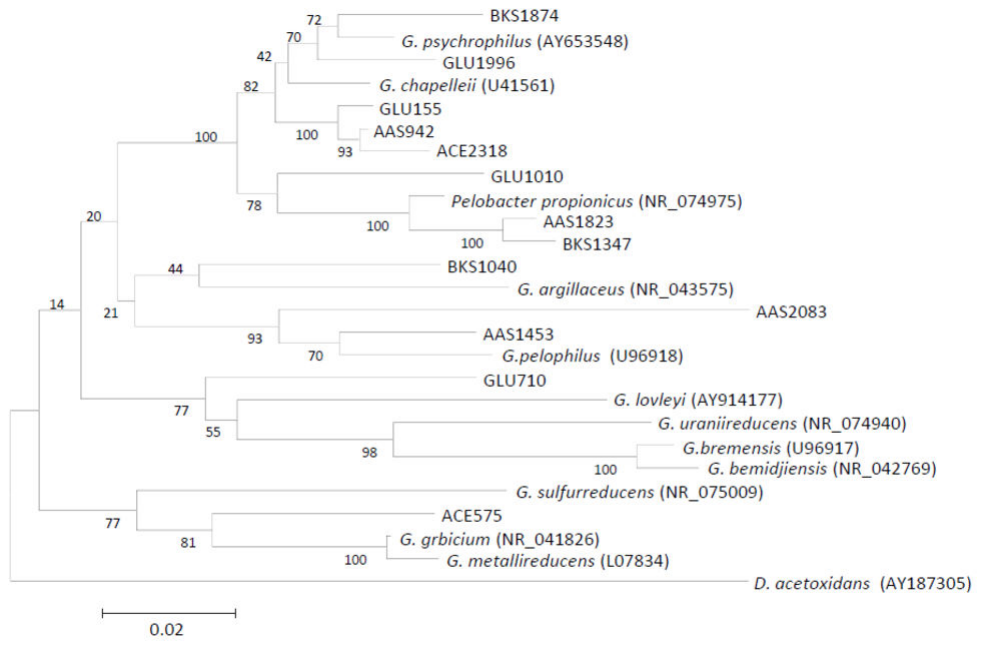
Neighbor-joining tree based on 16S rRNA gene sequences showing phylogenetic relationships in the genus *Geobacter*. Refer to Table 2 for the major OTUs. *Desulfuromonas acetoxidans* was used as an outgroup. Bootstrap values (100 trials, only > 50 are shown) are indicated at branching points. The bar indicates 2% sequence divergence. Accession numbers are shown in parentheses.

Analyses of sequences affiliated with the family *Geobacteraceae* identified several major OTUs (>5% of the total *Geobacteraceae* sequences) from the AM- and GM-anode biofilm samples (Table 2). Examination of the phylogenetic relationships among these *Geobacteraceae* OTUs and several reference strains revealed that the dominant *Geobacter* OTU in the AM-anode biofilm (OTU ACE575, 89%) was affiliated with a distinct cluster comprised of *G. grbicium*, *G. metallireducens*, and *G. sulfurreducens* (Figure 4 and Table 2). This phylogenetic group was previously referred to as the *G. metallireducens* clade [39]. Notably, however, this group of *Geobacter* OTUs was only present as minor components in the other MFC samples. Relatives of *G. sulfurreducens*, which is a well-characterized EAB that has high electricity-generating capacity in the presence of acetate [40], are frequently detected in acetate-fed bioelectrochemical systems [41-43]. Together, the present results suggest that these *Geobacter* strains are preferentially selected in anode biofilms when acetate is supplied as the sole substrate.

**Table 2:** Major OTUs affiliated with the family *Geobacteraceae*.

Major OTU^a^	No. of seq. (%)	Closest match 16S rRNA gene sequence (% identical)
Bulk soil		
BKS1040	22 (25)	*Geobacter argillaceus* NR_043575 (92%)
BKS1347	23 (26)	*Pelobacter propionicus* NR_074975 (97%)
BKS1874	22 (25)	*Geobacter psychrophilus* NR_043075 (98%)
Others	21 (24)	
Total	88 (100)	
Anode-associated soil		
AAS942	62 (20)	*Geobacter psychrophilus* NR_043075 (96%)
AAS1453	126 (40)	*Geobacter pelophilus* NR_026077 (96%)
AAS1823	43 (14)	*Pelobacter propionicus* NR_074975 (97%)
AAS2083	30 (10)	*Geobacter pelophilus* NR_026077 (91%)
Others	52 (16)	
Total	313 (100)	
AM-anode biofilm		
ACE575	290 (89)	*Geobacter grbicium* NR_041826 (95)
ACE2318	29 (9)	*Geobacter psychrophilus* NR_043075 (95)
Others	8 (2)	
Total	327 (100)	
GM-anode biofilm		
GLU155	304 (40)	*Geobacter psychrophilus* NR_043075 (97%)
GLU710	39 (5)	*Geobacter daltonii* NR_074916 (91%)
GLU1010	233 (30)	*Pelobacter propionicus* NR_074975 (94)
GLU1996	40 (5)	*Geobacter psychrophilus* NR_043075 (97%)
Others	164 (20)	
Total	780 (100)	

^a^ OTUs that include more than 5% of the total *Geobacteraceae* sequence tags.

In the other samples, OTUs related to *G. psychrophilus*, *P. propionicus*, and *G. pelophilus* were abundantly detected. In particular, many *Geobacter* OTUs were relatives of *G. psychrophilus* [44], including the most abundant and second most abundant *Geobacter* OTUs in the GM-anode biofilm (GLU155) and anode-associated soil (AAS942), respectively. Although *G. psychrophilus* relatives were also detected in the bulk soil, their relative abundances increased in concordance with the electric outputs. From these observations, we suggest that the major EAB occurring in the rhizosphere sMFC were similar to those in the glucose-fed MFC. 

### Metagenomic insights into anode-associated microbes

To further compare the microbiomes present in the above-mentioned anode-biofilm and anode associated-soil samples, metagenomes extracted from these samples were shotgun sequenced using the Illumina platform. This system facilitates relatively rapid and cost-effect deep genome sequencing, but can only provide a read length of 100 bp, which is markedly shorter than that generated by the 454 pyrosequencing platform (400 to 700 bp). However, in a previous study assessing the utility of the Illumina platform in metagenomic analysis [45], Illumina- and 454-based contigs were mapped to Sanger reads from the same samples, demonstrating that the short and long read-based assemblies have comparable accuracies (14.2 and 20.7 errors per megabase, respectively) [45].

In the present metagenomic analysis, a single lane of the Illumina Hiseq 2000 sequencer was used for the biofilm and soil samples, yielding 164 mega reads of 16.4 Gb in total (approximately 4 Gb for each sample). Numerical data for the metagenome sequencing, contig assembly, and gene prediction are summarized in Table 1. The number and length of contigs indicate that the metagenome reads from the biofilm samples were more deeply assembled in contigs than those from the soil samples, indicating that the degree of community diversity, which was estimated by the rarefaction analysis (Figure 3A), affected the contig assembly and subsequent analyses (e.g., average gene length).

Genes with BLAST hits to the NCBI-NR database (BLAST-hit genes) were subjected to MEGAN analysis to predict their taxonomic distribution (Figure S2). At the domain level, over 80% of genes were affiliated with the domain *Bacteria*, with *Archaea* only representing a minor proportion of genes (2% to 9%; Figure S2). At the phylum level, members of *Proteobacteria* were the most abundant in all samples (Table S2 and Figure S3); however, it is important to consider that this trend, at least partially, may reflect the large number of genome sequences available for this phylum. However, the metagenomic data can provide insight into the microbiomes associated with the different MFC anodes by comparing the percent values of different samples for a certain taxonomic group. For example, *Deltaproteobacteria* (including *Geobacter*) increased in the two anode-biofilm samples (Table S2), *Betaproteobacteria* (including *Comamonas*) increased in the AM-anode biofilm (Table S2), whereas *Firmicutes* (including *Clostridium*) increased only in the GM-anode biofilm (Table S2 and Figure S3). These profiles corresponded well to the 16S rRNA-based phylogenetic data described above (Table S1 and Figure 3B).

**Figure 5:**
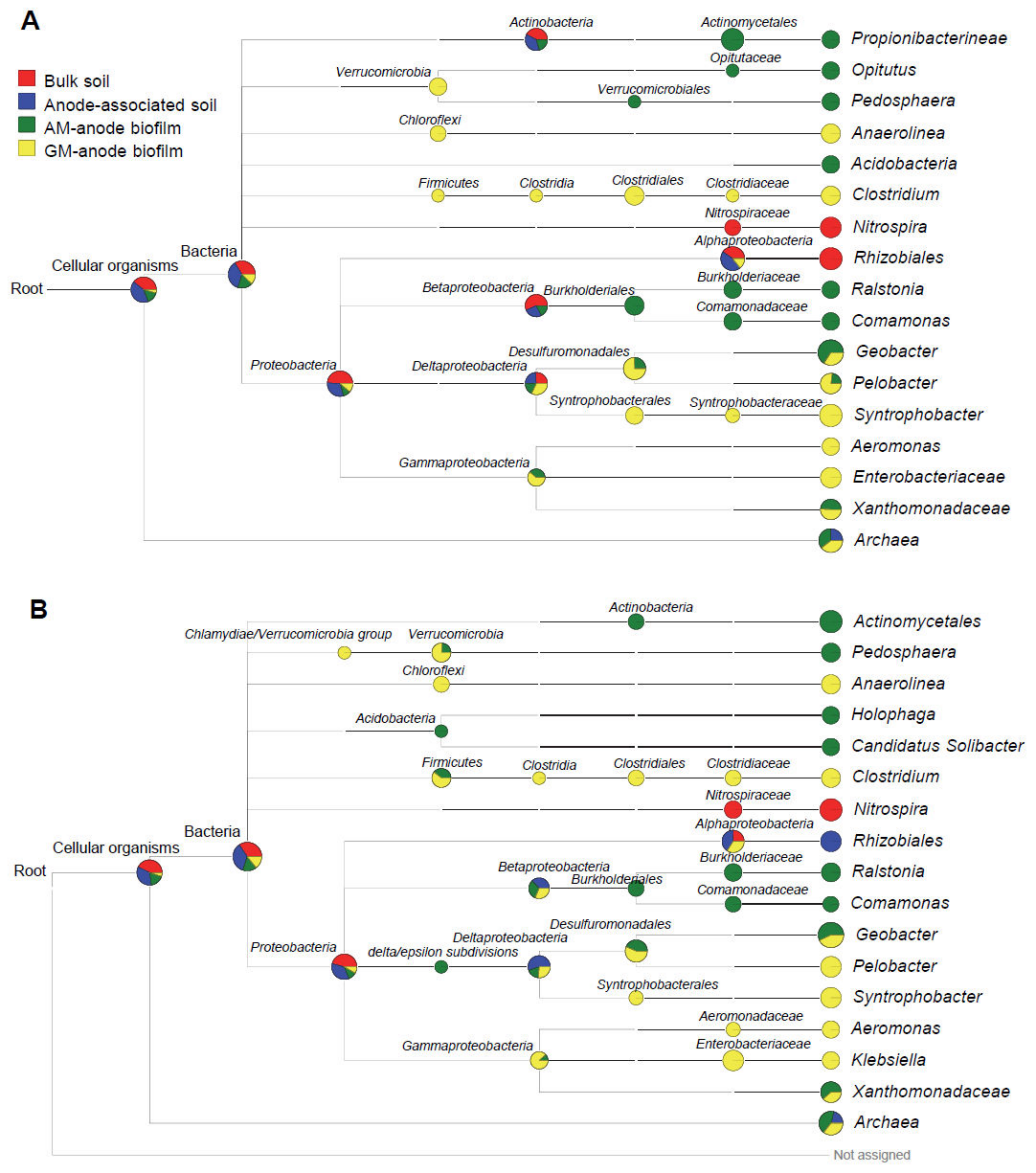
MEGAN trees constructed for genes with high BLAST scores assigned to the KEGG maps. (A) citrate cycle and (B) glycolysis/gluconeogenesis. Normalized numbers of genes assigned to each taxonomic group are presented in the comparative tree views of MEGAN. The size of each node is scaled logarithmically to indicate numbers of assigned genes in the four samples.

In the MFC systems analyzed in the present study, the glycolytic pathway and citrate cycle should be important for electricity generation. We therefore investigated the taxonomic distributions of genes that were assigned by MEGAN to the KEGG maps of the “citrate cycle” and “glycolysis/gluconeogenesis” (Figure 5). The constructed MEGAN trees show that abundantly represented taxa involved in these catabolic pathways, such as *Geobacter* in the anode-biofilm samples, *Comamonas* in the AM-abode biofilm, and *Clostridium* in the GM-anode biofilm, were also detected in the 16S rRNA gene-sequence analyses (Figure 3B). We also found that genes in the bulk and anode-associated soils were similarly distributed to the MEGAN trees, and many of the detected genes were assigned only to higher ranks, except for *Nitrospira* and *Rhizobiales*. This trend may be attributed to the short gene lengths for the soil samples (Table 1). In the GM-anode biofilm, fermentative bacteria, including *Anaerolinea* [46], *Clostridium* [33], and *Syntrophobacter* [47] were overrepresented, suggesting the importance of fermentative bacteria and their syntrophic associations with EABs in the conversion of glucose into electricity.

**Figure 6:**
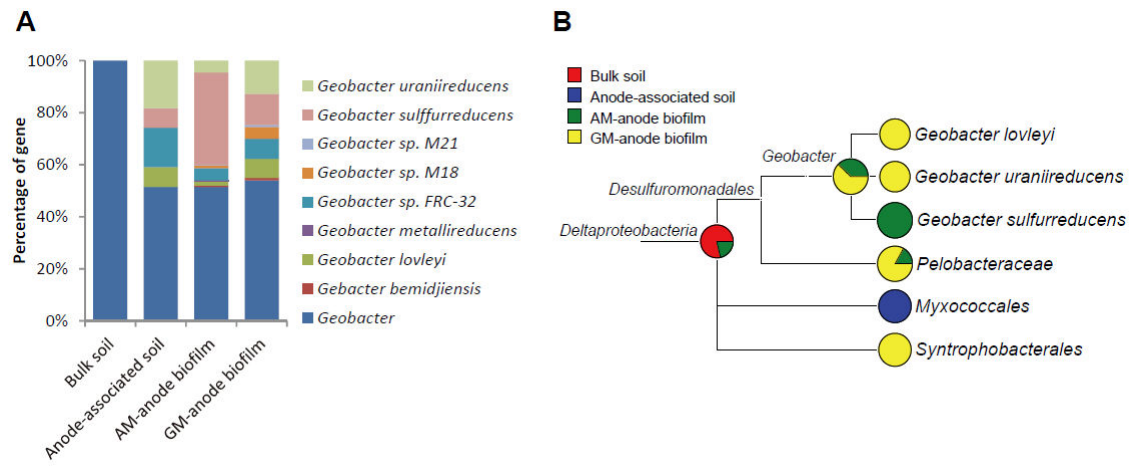
Metagenomic insights into the genus *Geobacter* obtained by MEGAN analyses. (A) Sub-genus level distribution of genes affiliated with the genus *Geobacter* based on BLAST homology. ‘*Geobacter*’ in the figures represents genes that were assigned to *Geobacter* at the genus level, but not to any genome-sequenced strain. (B) A partial MEGAN tree showing taxonomic distribution of genes encoding the acetate-catabolizing enzymes acetyl-CoA synthetase (EC 6.2.1.1), phosphate acetyltransferase (2.3.1.8), and acetate kinase (2.7.2.1), assigned to the class *Deltaproteobacteria*. LCA parameters: Min score, 50; top percent, 1.0; Min support, 2.

Genes assigned to the genus *Geobacter* based on BLAST homology were extracted, and we attempted to assign them to the eight genome-sequenced *Geobacter* strains (Figure 6A). The analysis showed that the *Geobacter* genes in the bulk soil could not be assigned to any strain, suggesting that the *Geobacter* strains in the bulk soil were not closely related to the eight fully sequenced stains. For the other samples, approximately half of the genes were not assigned to any isolated strain, while the remaining genes displayed characteristic distributions. Specifically, a high proportion (approximately 70%) of genes assigned to *G. sulfurreducens* was identified in the AM-anode biofilm, whereas the gene distribution pattern of the anode-associated soil was similar to that of the GM-anode biofilm. These results suggest that *Geobacter* EAB present in the anode-associated soil were similar to those in the GM-anode biofilm.

MEGAN was also used to assign acetate-catabolizing genes (acetyl-CoA synthetase, phosphate acetyltransferase, and acetate kinase) to taxa in the class *Deltaproteobacteria*, as *Geobacter* is known to preferentially utilize acetate as an electron donor for electricity generation [8,9] (Figure 6B). Acetate-catabolizing genes in the AM-anode biofilm were mostly assigned to *G. sulfurreducens*, whereas these genes in the GM-anode biofilm were related to *G. lovleyi* [48] and *G. uraniireducens* [49]. Unexpectedly, such genes in the anode-associated soil were assigned to the order *Mixococcales*. As the genomes of close relatives to *G. psychrophilus* have not yet been sequenced, the linkage of genes in the GM-anode biofilm and anode-associated soil to *G. psychrophilus* were not apparent. In addition, although clear differences in acetate-catabolizing genes between the AM- and GM-anode biofilms were observed (Figure 6B), genes assigned to the genus *Geobacter* was not detected in the anode-associated soil. This result may have resulted from the relatively small ratio of *Geobacter* to total bacteria and the insufficient metagenome dataset for analyzing genes at the genus level in the complex soil microbiome.

### Conclusions and perspectives

We conducted molecular phylogenetic and metagenomic analyses of the anode microbiomes of rhizosphere sMFCs to gain insight into EAB that generate electricity in these systems. In particular, we performed comparative analyses of suspected EAB affiliated with the family *Geobacteraceae* and found that possible EAB present in the anode-associated soil and GM-anode biofilm exhibited many similarities, including polarization behavior, 16S rRNA phylogeny, and metagenomic gene assignments. Together, these results suggest that these microbiomes generate electricity through similar mechanisms. A common feature of the anode-associated soil and GM-anode biofilm microbiomes that is distinct from the AM-anode biofilm microbiome is the occurrence of syntrophic interaction; in the rhizosphere and glucose-fed MFCs, EAB were considered to generate electricity from organics excreted by other organisms, such as rice plants in the rhizosphere sMFC and fermentative bacteria in the glucose-fed MFC (e.g., *Clostridium* abundantly detected in the GM-anode biofilm). Such syntrophy in these MFCs may promote the growth of certain electricity-producing *Geobacter*, such as *G. psychrophilus* relatives. In contrast, EAB related to *G. metallireducens* would preferentially grow in the acetate-fed MFC.

Our previous study found that rice plants excrete glucose and acetate as major root exudates, and that the supplementation of rhizosphere anodes with acetate enhances electricity generation from rhizosphere sMFCs [3]. These results suggest that acetate also served as the electron donor for EAB in the rhizosphere sMFC analyzed in the present study, and that the gradual supply of acetate from rice roots may facilitate the growth of specific *Geobacter* EAB. Evidence suggests that syntrophic interactions between EAB and other organisms are important for determining the electric output from MFCs [50,51]. It would therefore be interesting to further examine how EAB in the rhizosphere sMFC and glucose-fed MFC grow on anodes. To this end, electricity generation by closely related *Geobacter* isolates, including *G. psychrophilus*, will be investigated in both single cultures at different substrate concentrations and binary cultures with sugar-fermenting organisms. In addition, because several phylogenetically novel *Geobacter* sequence types were found in the present study, the isolation and physiological characterization of these species will provide valuable information regarding electricity-generating mechanisms in rhizosphere sMFCs.

## Supporting Information

Table S1 **Genus-level taxonomic groups detected by the pyrotag sequencing of 16S rRNA gene amplicons.**
(DOCX)

Table S2 **Phylum-level classification of genes with high BLAST scores assigned to bacteria.**
(DOCX)

Figure S1 **Time courses of cell voltages for the acetate-fed (**A**) and glucose-fed MFCs (**B**).** When the voltage was dropped down to 0.05 V, the electrolyte was changed to the fresh one containing the substrate (acetate or glucose, 0.5 g l^-1^) . The external resister was changed from 10,000 Ω to 1,000 Ω on day 20. On day 250, the operation was terminated, and DNA was extracted from anode biofilms.
(TIF)

Figure S2 **Comparisons in normalized numbers of genes with high BLAST scores in the domain-level classification.** Ratios in normalized numbers of BLAST-hit genes in each node are shown in comparative tree view of MEGAN. A size of each node is scaled logarithmically to indicate numbers of assigned genes. Numbers in brackets indicate percentages of sequences assigned to each node to total number of predicted genes for the bulk soil, anode-associated soil, AM-anode biofilm and GM-anode biofilm.
(TIF)

Figure S3 **Phylum-level classification of bacteria in the four samples.** (A) 16S rRNA gene sequences by the RDP classifier; (B) genes with high BLAST scores by the MEGAN LCA algorithm; (C) metagenome contigs by BLSOM analysis.
(TIF)
