# Supplementary material for: Comparative Metagenomics of Anode-Associated Microbiomes Developed in Rice Paddy-Field Microbial Fuel Cells
Source: PLoS One. 2013 Nov 1;8(11):e77443. doi: 10.1371/journal.pone.0077443 (PMC3815305; doi:10.1371/journal.pone.0077443)
Supplement: Table S1 — Genus-level taxonomic groups detected by the pyrotag sequencing of 16S rRNA gene amplicons. (DOCX) [file pone.0077443.s001.docx]

**TABLE S1** Genus-level taxonomic groups detected by the pyrotag sequencing of 16S rRNA gene amplicons^a^

| Taxonomic group | Bulk soil | Anode-associated soil | AM-anode biofilm | GM-anode biofilm |
| --- | --- | --- | --- | --- |
| *Acetanaerobacterium* | 0 | 0 | 4 | 3 |
| *Acetivibrio* | 12 | 24 | 22 | 41 |
| *Acidovorax* | 0 | 0 | 47 | 30 |
| *Aeromonas* | 1 | 0 | 0 | 7 |
| *Aminobacter* | 0 | 0 | 5 | 1 |
| *Anaerobacter* | 2 | 57 | 13 | 24 |
| *Anaerolinea* | 1 | 9 | 0 | 0 |
| *Anaeromyxobacter* | 39 | 73 | 11 | 0 |
| *Anaerosporobacter* | 2 | 3 | 0 | 7 |
| *Anaerovorax* | 7 | 15 | 109 | 41 |
| *Aquicella* | 5 | 1 | 0 | 0 |
| *Aquitalea* | 0 | 0 | 0 | 27 |
| *Azospirillum* | 1 | 0 | 1 | 8 |
| *Bacillariophyta* | 13 | 46 | 0 | 1 |
| *Bacillus* | 24 | 103 | 20 | 2 |
| *Bacteroides* | 0 | 0 | 0 | 13 |
| *Bellilinea* | 9 | 41 | 0 | 0 |
| *Bradyrhizobium* | 5 | 0 | 3 | 0 |
| *Brevibacillus* | 0 | 6 | 1 | 0 |
| *Burkholderia* | 0 | 1 | 5 | 3 |
| *Caldilinea* | 7 | 22 | 0 | 0 |
| *Caulobacter* | 9 | 4 | 2 | 0 |
| *Chitinophaga* | 1 | 0 | 12 | 40 |
| *Chlorophyta* | 2 | 12 | 0 | 0 |
| *Clostridium* | 30 | 140 | 92 | 1819 |
| *Cohnella* | 2 | 5 | 4 | 0 |
| *Comamonas* | 0 | 0 | 966 | 187 |
| *Cupriavidus* | 0 | 0 | 4 | 4 |
| *Defluviicoccus* | 1 | 7 | 0 | 0 |
| *Dehalogenimonas* | 8 | 14 | 0 | 0 |
| *Delftia* | 0 | 0 | 18 | 0 |
| *Desulfatirhabdium* | 0 | 11 | 0 | 0 |
| *Desulfobacca* | 2 | 12 | 0 | 0 |
| *Desulfobulbus* | 0 | 9 | 0 | 0 |
| *Desulfovibrio* | 0 | 0 | 4 | 2 |
| *Diaphorobacter* | 0 | 0 | 8 | 0 |
| *Dokdonella* | 0 | 0 | 8 | 3 |
| *Filimonas* | 1 | 0 | 50 | 1 |
| *Flavobacterium* | 3 | 0 | 5 | 0 |
| *Gemmatimonas* | 8 | 21 | 0 | 0 |
| *Geobacter* | 55 | 209 | 327 | 761 |
| *Geothrix* | 20 | 37 | 197 | 155 |
| Gp1 | 30 | 184 | 2 | 3 |
| Gp11 | 40 | 139 | 0 | 0 |
| Gp16 | 8 | 8 | 3 | 1 |

**TABLE S1** Continued.

| Taxonomic group | Bulk soil | Anode-associated soil | AM-anode biofilm | GM-anode biofilm |
| --- | --- | --- | --- | --- |
| Gp17 | 12 | 11 | 0 | 0 |
| Gp18 | 15 | 79 | 1 | 0 |
| Gp22 | 7 | 24 | 0 | 0 |
| Gp25 | 8 | 37 | 0 | 0 |
| Gp3 | 0 | 4 | 26 | 4 |
| Gp4 | 37 | 85 | 1 | 2 |
| Gp5 | 27 | 98 | 0 | 0 |
| Gp6 | 129 | 408 | 4 | 1 |
| Gp7 | 30 | 70 | 1 | 0 |
| *Haliscomenobacter* | 2 | 12 | 0 | 0 |
| *Hydrogenoanaerobacterium* | 0 | 0 | 23 | 0 |
| *Hyphomicrobium* | 5 | 12 | 1 | 1 |
| *Kaistia* | 0 | 8 | 5 | 1 |
| *Klebsiella* | 0 | 0 | 0 | 15 |
| *Kofleria* | 5 | 0 | 0 | 0 |
| *Ktedonobacter* | 7 | 44 | 0 | 0 |
| *Longilinea* | 12 | 11 | 0 | 1 |
| *Lutispora* | 1 | 4 | 109 | 0 |
| *Methylocystis* | 15 | 33 | 0 | 1 |
| *Methylomonas* | 5 | 17 | 0 | 0 |
| *Niabella* | 0 | 4 | 5 | 0 |
| *Niastella* | 8 | 46 | 0 | 0 |
| *Nitrospira* | 58 | 121 | 6 | 4 |
| *Ochrobactrum* | 0 | 0 | 15 | 0 |
| OD1 | 51 | 142 | 0 | 0 |
| OP10 | 12 | 16 | 0 | 0 |
| OP11 | 1 | 19 | 0 | 0 |
| *Opitutus* | 2 | 0 | 1 | 73 |
| *Oscillibacter* | 0 | 0 | 2 | 1 |
| *Oxobacter* | 1 | 0 | 0 | 9 |
| *Paenibacillus* | 11 | 55 | 2 | 1 |
| *Pelobacter* | 2 | 10 | 34 | 253 |
| *Phenylobacterium* | 3 | 14 | 0 | 0 |
| *Pleomorphomonas* | 0 | 0 | 0 | 8 |
| *Propionivibrio* | 0 | 0 | 54 | 26 |
| *Proteiniborus* | 0 | 0 | 19 | 0 |
| *Pseudomonas* | 0 | 10 | 210 | 11 |
| *Ralstonia* | 0 | 0 | 5 | 2 |
| *Rhodocyclus* | 0 | 0 | 8 | 1 |
| *Rhodoplanes* | 9 | 8 | 1 | 0 |
| *Sarcina* | 1 | 9 | 1 | 26 |
| *Sedimentibacter* | 0 | 0 | 7 | 2 |
| *Smithella* | 13 | 53 | 1 | 1 |
| *Spartobacteria* | 1 | 29 | 0 | 10 |
| *Sporomusa* | 3 | 11 | 5 | 251 |

**TABLE S1** Continued.

| Taxonomic group | Bulk soil | Anode-associated soil | AM-anode biofilm | GM-anode biofilm |
| --- | --- | --- | --- | --- |
| *Sporotalea* | 7 | 46 | 25 | 26 |
| *Stenotrophomonas* | 0 | 0 | 15 | 0 |
| Subdivision3 | 2 | 31 | 0 | 21 |
| *Syntrophorhabdus* | 8 | 33 | 2 | 0 |
| *Telmatospirillum* | 8 | 4 | 1 | 0 |
| *Terrimonas* | 10 | 20 | 12 | 0 |
| *Tissierella* | 0 | 0 | 107 | 1 |
| TM7 | 4 | 0 | 0 | 0 |
| *Tumebacillus* | 7 | 9 | 0 | 0 |
| WS3 | 12 | 20 | 0 | 0 |
| *Zoogloea* | 0 | 0 | 2 | 12 |
| Others | 1358 | 3408 | 1795 | 1542 |
| total | 2257 | 6288 | 4449 | 5491 |

^a^ Sequences were classified using the RDP classifier, and taxonomic groups containing more than 3 sequences are shown.
