# Supplementary material for: Comparative Metagenomics of Anode-Associated Microbiomes Developed in Rice Paddy-Field Microbial Fuel Cells
Source: PLoS One. 2013 Nov 1;8(11):e77443. doi: 10.1371/journal.pone.0077443 (PMC3815305; doi:10.1371/journal.pone.0077443)
Supplement: Table S2 — Phylum-level classification of genes with high BLAST scores assigned to bacteria. (DOCX) [file pone.0077443.s002.docx]

**TABLE S2** Phylum-level classification of the BLAST-hit genes assigned to bacteria

| Taxonomic group | Bulk soil | | Anode-associated soil | | AM-anode biofilm | | GM-anode biofilm | |
| --- | --- | --- | --- | --- | --- | --- | --- | --- |
|  | Reads | Percent | Reads | Percent | Reads | Percent | Reads | Percent |
| *Actinobacteria* | 196 | 2.5 | 280 | 2.5 | 3108 | 8.7 | 413 | 1.2 |
| *Aquificae* | 0 | 0.0 | 8 | 0.1 | 5 | 0.0 | 0 | 0.0 |
| Bacteroidetes/Chlorobi group | 89 | 1.1 | 235 | 2.1 | 418 | 1.2 | 188 | 0.5 |
| *- Bacteroidetes* | 64 | 0.8 | 80 | 0.7 | 331 | 0.9 | 154 | 0.4 |
| *- Chlorobi* | 0 | 0 | 10 | 0.1 | 36 | 0.1 | 26 | 0.1 |
| *- Inganvibacteria* | 18 | 0.2 | 125 | 1.1 | 13 | 0.0 | 6 | 0.0 |
| Chlamydiae/Verrucomicrobia group | 42 | 0.5 | 56 | 0.5 | 1420 | 4.0 | 1194 | 3.4 |
| *- Chlamydiae* | 0 | 0 | 0 | 0.0 | 8 | 0.0 | 0 | 0.0 |
| *- Lentisphaerae* | 0 | 0 | 0 | 0.0 | 5 | 0.0 | 5 | 0.0 |
| *- Verrucomicrobia* | 41 | 0.5 | 54 | 0.5 | 1404 | 3.9 | 1185 | 3.3 |
| *Chloroflexi* | 75 | 1.0 | 109 | 1.0 | 97 | 0.3 | 1111 | 3.1 |
| *Cyanobacteria* | 32 | 0.4 | 51 | 0.5 | 92 | 0.3 | 119 | 0.3 |
| *Deferribacteres* | 0 | 0 | 0 | 0.0 | 0 | 0.0 | 6 | 0.0 |
| *Deinococcus-Thermus* | 7 | 0.1 | 13 | 0.1 | 17 | 0.0 | 21 | 0.1 |
| Environmental samples | 35 | 0.4 | 41 | 0.4 | 37 | 0.1 | 29 | 0.1 |
| Fibrobacteres/Acidobacteria group | 122 | 1.5 | 96 | 0.9 | 2451 | 6.9 | 106 | 0.3 |
| *- Acidobacteria* | 121 | 1.5 | 96 | 0.9 | 2449 | 6.9 | 12 | 0.0 |
| *Firmicutes* | 63 | 0.8 | 93 | 0.8 | 500 | 1.4 | 2443 | 6.9 |
| *Fusobacteria* | 0 | 0 | 0 | 0.0 | 0 | 0.0 | 5 | 0.0 |
| *Gemmatimonadetes* | 33 | 0.4 | 8 | 0.1 | 12 | 0.0 | 0 | 0.0 |
| *Nitrospirae* | 482 | 6.1 | 240 | 2.2 | 19 | 0.1 | 12 | 0.0 |
| *Planctomycetes* | 33 | 0.4 | 42 | 0.4 | 126 | 0.4 | 86 | 0.2 |
| *Proteobacteria* | 3881 | 49.2 | 5410 | 49.2 | 17043 | 47.7 | 20541 | 57.8 |
| *- Alphaproteobacteria* | 1225 | 15.5 | 2382 | 21.7 | 452 | 1.3 | 1796 | 5.0 |
| *- Betaproteobacteria* | 946 | 12.0 | 703 | 6.4 | 6193 | 17.3 | 1944 | 5.5 |
| - delta/epsilon subdivision | 285 | 3.6 | 471 | 4.3 | 7119 | 19.9 | 9868 | 27.7 |
| *- Deltaproteobacteria* | 282 | 3.6 | 468 | 4.3 | 7105 | 19.9 | 9845 | 27.7 |
| *- Epsilonproteobacteria* | 0 | 0 | 0 | 0.0 | 9 | 0.0 | 20 | 0.1 |
| *- Gammaproteobacteria* | 181 | 2.3 | 405 | 3.7 | 1695 | 4.7 | 5171 | 14.5 |
| *Spirochaetes* | 9 | 0.1 | 8 | 0.1 | 37 | 0.1 | 54 | 0.2 |
| *Synergistetes* | 0 | 0 | 5 | 0.0 | 15 | 0.0 | 12 | 0.0 |
| *Thermotogae* | 0 | 0 | 0 | 0.0 | 13 | 0.0 | 25 | 0.1 |
| Unclassified bacteria | 1710 | 25.0 | 2446 | 26.8 | 4089 | 13.9 | 3776 | 12.5 |
| Total bacteria | 6839 | 86.7 | 9141 | 83.2 | 29499 | 82.6 | 30141 | 84.7 |
