# Supplementary figures and images for: Comparative Metagenomics of Anode-Associated Microbiomes Developed in Rice Paddy-Field Microbial Fuel Cells
Source: PLoS One. 2013 Nov 1;8(11):e77443. doi: 10.1371/journal.pone.0077443 (PMC3815305; doi:10.1371/journal.pone.0077443)

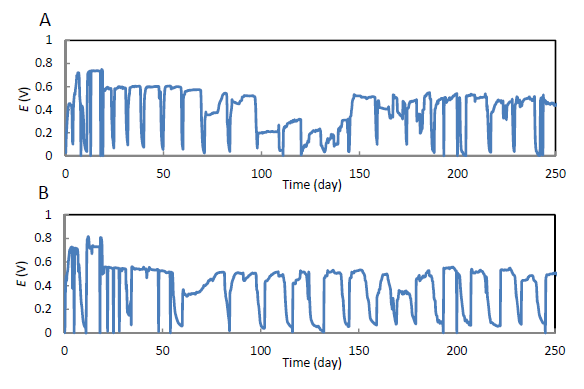

Supplement: Figure S1 — Time courses of cell voltages for the acetate-fed (A) and glucose-fed MFCs (B). When the voltage was dropped down to 0.05 V, the electrolyte was changed to the fresh one containing the substrate (acetate or glucose, 0.5 g l-1) . The external resister was changed from 10,000 Ω to 1,000 Ω on day 20. On day 250, the operation was terminated, and DNA was extracted from anode biofilms. (TIF) [file pone.0077443.s003.tif]

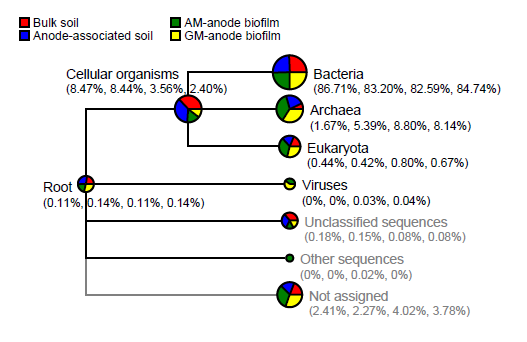

Supplement: Figure S2 — Comparisons in normalized numbers of genes with high BLAST scores in the domain-level classification. Ratios in normalized numbers of BLAST-hit genes in each node are shown in comparative tree view of MEGAN. A size of each node is scaled logarithmically to indicate numbers of assigned genes. Numbers in brackets indicate percentages of sequences assigned to each node to total number of predicted genes for the bulk soil, anode-associated soil, AM-anode biofilm and GM-anode biofilm. (TIF) [file pone.0077443.s004.tif]

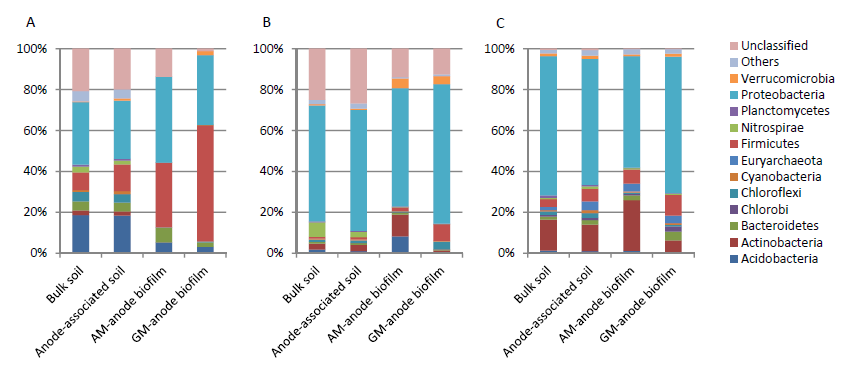

Supplement: Figure S3 — Phylum-level classification of bacteria in the four samples. (A) 16S rRNA gene sequences by the RDP classifier; (B) genes with high BLAST scores by the MEGAN LCA algorithm; (C) metagenome contigs by BLSOM analysis. (TIF) [file pone.0077443.s005.tif]
